# The International Medical Annual

**Published:** 1897-04

**Authors:** 


The International Medical Annual and Practitioner's Index: A work of reference for medical practitioners. 1897, fifteenth year. New York, E. B. Treat, 241 and 243 West Twenty-third Street; Chicago, 199 Clark Street. Price, $2.75.

The annual issues of this excellent book have been regularly noticed in these pages as they have appeared. If a practitioner would have a concise and yet comprehensive review of the changes in views and of practice in any particular department of medicine for the last fifteen years, let him look over the different issues of this book. He will lose no time in making this retrospect since each issue, as has been repeatedly stated in our reviews, is provided with a copious index. In the present volume this index occupies nineteen pages, double column, small type.

The first ninety pages constitute a dictionary of new remedies. Here we find short and pithy treatises on all the new medicines of every kind, the coal tar derivatives occupying the largest place. Among these new remedies may be found the familiar drug of our own materia medica, Apocynum-cannabinum, which is found useful in dropsy, especially in cardiac troubles.

Another remedy is Extract of the Ovary for amenorrhea. Serpent venom for protection against serpent bites. Supra-renal capsule extract for Addison's disease. Thyroid Extract for goitre, myxedema, and obesity. Urea for increase of urine, especially in connection with cirrhosis of the liver. Indeed, there is a special chapter on this subject entitled "Glandular Therapeutics."

Rhus-toxicodendron is another remedy introduced, though apparently but little use is made of it Yet another familiar remedy is Sambucus-niger, which is used for general anasarca, pulmonary edema, and ascites due to subacute nephritis.

In the chapter on "New Treatment," Viburnum-prunifolium is used for abortion and Hydrastis for uterine hemorrhage. This will hardly be news to the homeopathist, but it is significant of the trend of medical thought and of the value of this book in displaying this trend.

In the treatment of bronchitis, it is observed that "each individual case demands its own special investigation and a line of treatment in accordance with the results of this investigation." Poultices in chest affections are denounced as being "of little or no value in the treatment of chest affections." "To watch a small child with extensive broncho-pneumonia fighting for breath, and then to further hamper its efforts by ordering poultices weighing about a pound seems hardly a scientific procedure." Over-feeding in these cases is also deprecated, for two reasons: "the burden arising from undigested food in the stomach, and there is also the risk of loading the blood with more nutritive material than the imperfect respiration can act upon in the process of sanguinification."

For burns Picric-acid is recommended. "The burn is dressed with a lint dipped in a saturated solution of Picric-acid, and then a layer of absorbent cotton is applied over the lint." Meanwhile Dr. A. J. Hall draws attention to the dangers of treating extensive burns with Boracic ointment, as it is known to develop "an extensive erythematous eruption, which may be followed by death. At the necropsy nothing was found to account for death, and previous to the eruption the boy was doing well."

In the treatment of Collapse *Camphor* is recommended. Also placing the head lower than the body, active rubbing, hypodermic injections of Ether and Caffein, enemas of Brandy or Champagne, whiffs of Nitrite of Amyl, slapping the face with a wet towel, Laborde's rhythmic tractions of the tongue, inhalations of Oxygen, and artificial respiration.

There is an interesting article upon the occurrence of loose bodies in the joints, and also a comment upon inflammation of the semilunar cartilages of the knee-joint. Treatment by rest has no effect and massage is recommended. The writer of this review had a case of this kind which he cured in a short time with homeopathic remedies after it had persisted under other treatment for a whole year.

In speaking of the Larynx a case is described aided by a photographic illustration, showing how, by means of the Roentgen rays, a lost intubation tube was located in the trachea.

Fractures of the patella are treated by wiring the fragments together subcutaneously. The results are very satisfactory. This method is, however, only useful for very recent fractures.

Whooping Cough is considered, and the infecting agent is now considered to be, not the bacillus found in the sputum but an ameba having "a finely granular protoplasm and great capability of movement." It is to be treated antiseptically and the Hydrozone of Charles Marchand, declared to be the best antiseptic. Other remedies of an antiseptic character are mentioned, but Peroxide of Hydrogen, or better still Hydrozone, Marchand, is at the head of the list.

The article on Phthisis is very full and the reviewer has not time or space to do full justice to it. He, therefore, passes it over with this brief reference.

The foregoing is but a slight reference to the many interesting articles in this book. It is impossible to do justice to them and so the reviewer quits the subject. In closing he desires to draw attention to the many illustrations, colored and photographic, which further elucidate the text, and to the alphabetical arrangement with leading words at the top of the page as in a dictionary, which, together with the full index, renders it easy to find any desired subject instantly.

